# AI Therapy Chatbots in Youth With Attention-Deficit/Hyperactivity Disorder: A Nurse-Led, Risk-Tiered Policy Framework

**DOI:** 10.2196/86237

**Published:** 2026-09-15

**Authors:** Saeko Ishibashi, Kazumi Kubota

**Affiliations:** 1 School of Nursing, Sapporo City University, Hokkaido, Japan; 2 Shimonoseki City University, 2-1-1 Daigaku-cho, Yamaguchi, 751-8510, Japan, 81 83-252-0288; 3 Department of Healthcare Information Management, University of Tokyo Hospital, Tokyo, Japan; 4 National Center for Child Health and Development, Tokyo, Japan

**Keywords:** attention-deficit/hyperactivity disorder, ADHD, therapy chatbot, virtual companion, nursing informatics, risk‑tiered regulation, child safety, real‑world evidence, artificial intelligence, AI

## Abstract

AI-powered therapy chatbots and virtual companions are beginning to enter routine mental health care, yet safeguards for children and adolescents remain underdeveloped. For youth with attention-deficit/hyperactivity disorder (ADHD), these tools may offer brief coaching, psychoeducation, and structured between-visit check-ins across clinic, school, and home settings, but they also raise concerns about inappropriate content, overreliance, quiet model drift, privacy across health and education systems, and missed crisis escalation. In this Viewpoint, we propose a nurse-led, risk-tiered policy and implementation framework for integrating AI therapy chatbots into youth ADHD care safely, effectively, and equitably. The framework has 3 linked layers: policy levers, implementation enablers, and measurable outcomes. We emphasize child-specific safeguards, predetermined change control for adaptive models, equity-oriented procurement and reimbursement, interoperability and safety reporting, and nurse-led human-in-the-loop oversight. We further clarify that nursing-generated data—such as structured assessments, triage actions, escalation records, and supervision workload—can function both as governance inputs and as outcome-relevant signals within monitoring and service evaluation. We also outline a phased implementation road map and a concise indicator set that schools and clinics can monitor without major new infrastructure. A nurse-led, risk-tiered approach, combined with age-appropriate safeguards, equity-by-design implementation, and privacy-preserving real-world learning, can help health systems and schools move from ad hoc adoption toward safer and more equitable use of AI therapy chatbots for youth with ADHD while the evidence base continues to mature.

## Introduction

### Background

Attention-deficit/hyperactivity disorder (ADHD) is common and costly, with functional impact across home, school, and community contexts throughout development [1-4]. Clinical guidelines recommend multimodal treatment—behavioral interventions, supports in school, and medication when appropriate [1,2]. Capacity constraints and unequal access, however, remain stubborn problems for families. In parallel, AI-powered therapy chatbots and virtual companions have advanced quickly. Early studies suggest that conversational agents can be feasible and may offer short-term symptom benefits in adjacent populations and indications (eg, student and young adult samples and non-ADHD conditions) [5]. However, direct evidence for child- and ADHD-specific applications is still limited and uneven, and real-world workflows and safety reporting remain underdeveloped—one reason why we argue that governance and safety guardrails matter now, not later. These gaps motivate our implementation-focused framework.

Conversational agents are already reaching families. The more urgent question is how to use them safely and equitably and how to fit them into real clinic and school workflows without creating new risks—especially for minors with ADHD, who may be more sensitive to design choices that affect attention, impulse control, and time on task. Regulatory and standard bodies have issued broad guidance on AI ethics and risk management [6-9], and there is growing clarity on good machine learning practice and predetermined change control plans (PCCPs) for adaptive software [7,8]. However, concrete, child-specific guardrails and the day-to-day implementation infrastructure across clinics and schools remain incomplete. Nurses—particularly school nurses and care managers—are well positioned to provide human-in-the-loop oversight; integrate digital tools into measurement-based, stepped care pathways; and monitor both outcomes and safety [10-12]. Therefore, this Viewpoint offers a pragmatic governance and implementation framework tailored to therapy chatbots in ADHD care.

### Aim of This Viewpoint

This Viewpoint aimed to propose a nurse-led, risk-tiered policy and implementation framework for integrating AI-powered therapy chatbots into youth ADHD care across clinic, school, and home settings. Our goal is not to endorse a specific product but to define near-term steps that health systems, schools, payers, and developers can take together.

### Ethical Considerations

This Viewpoint did not involve human participants or identifiable data.

## Why Therapy Chatbots for ADHD? What Could Go Wrong?

The promise is easy to understand. A conversational tool can help youth practice organization and time management skills, support caregivers with brief coaching ideas, and collect structured check-ins that can feed measurement-based care between visits. For families facing long waits, transportation barriers, or stigma, a low-friction tool can feel like a practical bridge rather than a replacement for care.

The risks are just as real, especially for minors. Without child-appropriate design and clear boundaries, chatbots can produce inaccurate or inappropriate content; blur the line between support and dependence; or encourage patterns of use that displace sleep, schoolwork, or offline coping. In ADHD in particular, engagement features that resemble rewards can unintentionally reinforce “more time on the app” even when that is not clinically helpful. The most serious risk is failure to recognize and escalate crisis signals such as self-harm language, threats of harm to others, abuse disclosures, or severe distress. Another risk is quiet model drift: adaptive updates can change performance over time, creating safety or equity problems that remain invisible unless they are monitored. Finally, youth ADHD care often crosses health and education settings, where privacy and consent rules differ; unless governance is explicit, well-intentioned integration can become legally and ethically fragile. These realities are why we argue for a nurse-led, risk-tiered approach that matches oversight and evaluation to intended use and potential impact.

## A Nurse-Led, Risk-Tiered Governance Framework

**Figure 1.**
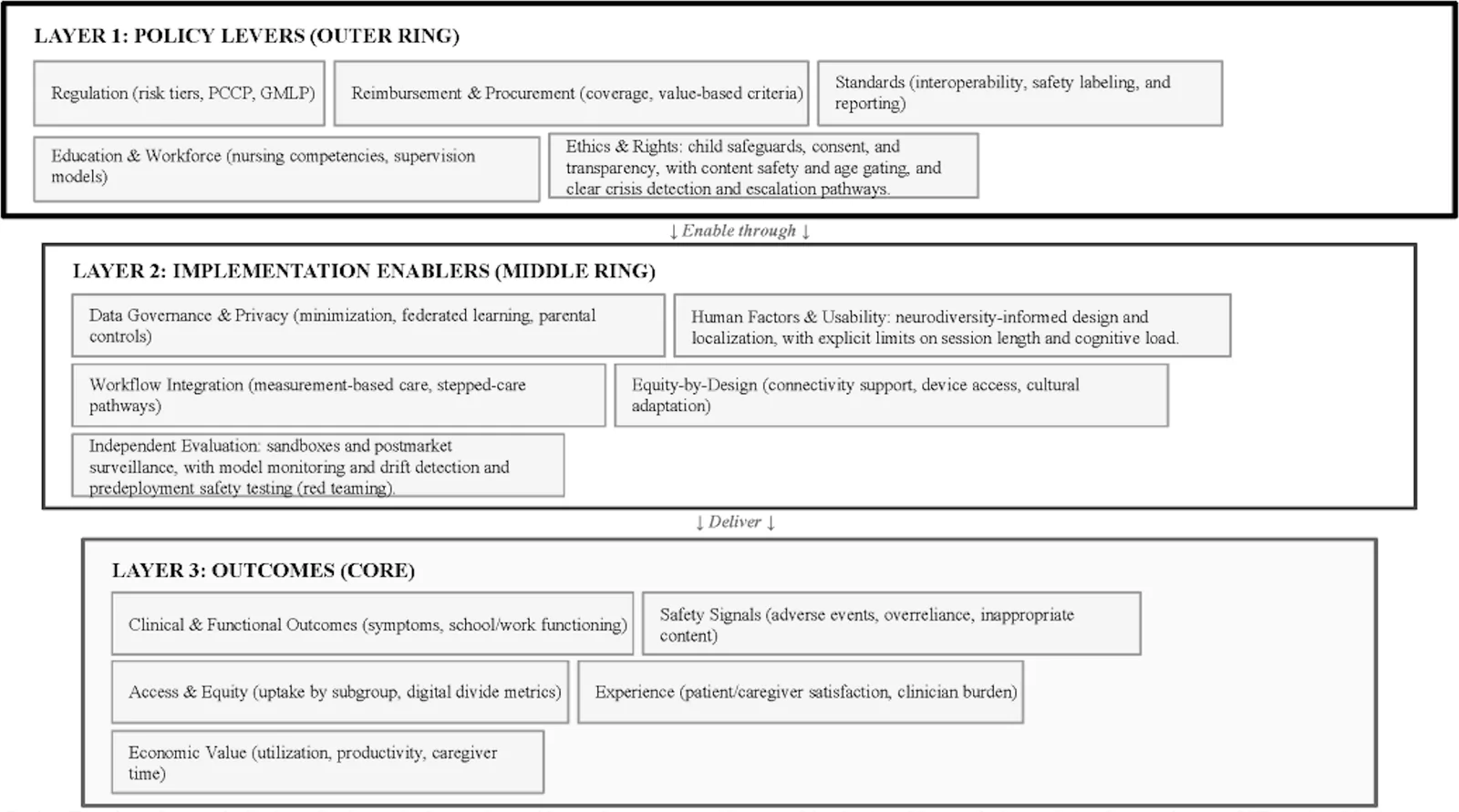
Nurse-led, risk-based governance framework for integrating AI into attention-deficit/hyperactivity disorder care across the lifespan. This framework aligns policy levers (regulation with risk tiers, predetermined change control plans [PCCPs], and good machine learning practice [GMLP]; reimbursement and procurement; standards for interoperability, safety labeling, and reporting; ethics and rights; and workforce development) with implementation enablers (data governance and privacy; human factors and usability—including limits on session length and cognitive load; workflow integration; equity by design; and independent evaluation with model monitoring, drift detection, and predeployment red teaming) and outcomes (access and equity, safety signals, clinical and functional outcomes, experience, and economic value). Nursing leadership provides human-in-the-loop oversight across clinic, school, community, and home settings, and nursing-generated data (eg, assessments, triage actions, escalation logs, and supervision workload) function as both governance inputs and outcome-relevant signals for ongoing learning.

Figure 1 summarizes a 3-layer framework that aligns policy levers, implementation enablers, and outcomes to guide responsible adoption of therapy chatbots and virtual companions in youth ADHD services. We start with what needs to be set at the system level, move to what must be in place for safe day-to-day use, and end with a small set of outcomes that keep performance visible rather than assumed. Nursing leadership is positioned as the practical anchor for human-in-the-loop oversight across clinic, school, community, and home settings, with clear coordination rather than replacement of interdisciplinary clinical and educational expertise. In this framework, nursing-generated data are not treated as passive documentation alone. Structured nursing observations, triage actions, escalation records, follow-up contacts, and supervision workload can serve as governance inputs and as outcome-relevant signals for safety oversight, resource planning, and service evaluation.

At the policy level, risk-tiered regulation and change control matter because many AI-enabled tools evolve through updates; PCCPs help make changes transparent, testable, and reversible when needed. Financing and procurement then determine whether safe implementation is feasible in routine care. When nurse time for triage, coaching, monitoring, and escalation is treated as “extra,” integration becomes fragile and inequitable; when it is recognized and supported, supervision becomes consistent and sustainable. Standards and ethics also need to be practical. For youth-facing tools, this means child-appropriate transparency, content safety and age gating, clear limits that prevent harmful engagement patterns, and escalation pathways that reliably connect a young person with human support when risk emerges.

On the implementation side, governance should follow a minimization mindset, use privacy-preserving approaches when possible, and make role-based access and auditability routine. Human factors are particularly important in ADHD because design choices can affect attention, impulse control, and time on task. Tools should be co-designed with neurodiverse youth and caregivers, match language and reading level, and include clear limits on session length and frequency so that engagement does not become a proxy for benefit. Workflow integration is equally critical: chatbots should feed measurement-based and stepped care pathways with defined handoffs rather than creating parallel “shadow care.” Equity by design should be operational from the start, including expectations for low-bandwidth or offline-capable use where needed and realistic pathways for device or connectivity support through schools, community partners, or payers. Independent evaluation completes the picture. A combination of sandbox pilots, safety testing before scale-up, and postdeployment monitoring for drift and adverse events helps prevent silent failures that only become visible after harm accumulates.

Finally, outcomes should extend beyond symptom scores alone. Programs should monitor access and equity, safety signals (including inappropriate content and escalation performance), clinical and functional outcomes (including school functioning where feasible), experience for youth and caregivers as well as staff supervision burden, and economic value. A feasible indicator set—reported regularly and interpreted with subgroup lenses—creates a feedback loop that can inform procurement, reimbursement, and change control as real-world evidence (RWE) accumulates [13-16].

## Comparative Policy Options

Table 1 summarizes 5 policy options that are often discussed as separate levers—regulation, procurement, reimbursement, and evaluation infrastructure—but that tend to work best as a package. The goal here is not to argue for a single “best” approach; rather, the table makes trade-offs explicit so readers can match governance choices to local capacity, risk tier, and equity priorities.

In practice, settings with limited regulatory or technical capacity can still start by raising the procurement floor and clarifying the human-in-the-loop workflow while building toward stronger change control and cross-site learning over time.

**Table 1.** Policy options for integrating AI therapy chatbots into youth attention-deficit/hyperactivity disorder (ADHD) care.

Option	Primary lever	Description	Benefits	Risks and limits	Feasibility	Equity impact
Status quo+industry self-regulation	Market self-regulation	Voluntary standards and vendor-led safeguards, with existing device and consumer protection rules applied when applicable	Fast deployment with minimal administrative burden	Inconsistent evidence and safety reporting, uneven child safeguards, and limited accountability for drift and subgroup harms	High	Often negative to neutral unless paired with access supports and monitoring
Risk-tiered regulation with PCCPs^a^ for adaptive AI	Regulations and standards	Classify tools by intended use and risk tier and require transparency artifacts and PCCPs for adaptive updates	Improves transparency and change governance, supports drift management, and strengthens minor-specific safeguards	Requires regulatory capacity and may increase compliance costs and slow down iteration	Medium	Neutral to positive if fairness audits and subgroup performance reporting are required
Voluntary certification+public procurement criteria	Certification and procurement	Independent certification and buyer criteria for schools and clinics covering age-appropriate design, content moderation, transparency, and safety reporting	Scales minimum quality expectations and helps purchasers compare products	Variable uptake and risk of checkbox compliance or certification capture	Medium-high	Positive if criteria include accessibility, language support, and digital divide mitigation
Reimbursement for nurse-delivered digital ADHD care	Payment	Reimburse nurse-led triage, coaching, monitoring, and escalation work when using approved chatbot-supported workflows	Aligns incentives for safe integration and supports workforce readiness and supervision time	Requires payer alignment, guardrails, and documentation to prevent overuse and substitution for needed in-person care	Medium	Positive if paired with coverage for connectivity and device support and culturally responsive services
Federated RWE^b^ registry and safety surveillance	Data governance and evaluation	Privacy-preserving cross-site evaluation using a shared harm taxonomy, common standardized data elements (including nursing and escalation data), and federated protocols where data remain local and only aggregated insights are shared	Enables continuous learning, consistent safety signal detection, and cross-site analysis of nursing-sensitive oversight indicators without centralizing sensitive data	Setup complexity and requires governance agreements, technical tooling, and sustained funding	Medium	Positive if inclusive by design and if low-resource sites are supported to participate

a PCCP: predetermined change control plan.

b RWE: real-world evidence.

## Implementation Road Map

In low-resource school districts and community clinics, the road map should be treated as modular rather than all-or-nothing. A workable starting point is to define intended use and risk tier, assign a named human-in-the-loop lead with a clear escalation pathway, and track a small set of safety and outcome indicators; more complex elements such as federated evaluation infrastructure can be added later as staffing, governance capacity, and partnerships grow.

### Preparation (0-3 Months)

A steering group spanning nursing, child psychiatry, pediatrics, school health, caregivers, students, regulators, payers, and developers should be convened. Risk tiers and intended use statements for therapy chatbots in ADHD should be agreed upon. A minimal outcome set and a safety signal set that includes inappropriate content, missed escalations, and adverse events should be specified. Procurement criteria modeled on existing frameworks that cover safety, security, interoperability, clinical safety, and usability should be selected [17-20].

### Pilot and Sandbox (3‐9 Months)

School and clinic pilots with informed consent or assent, role-based human oversight (nurses as primary supervisors), and prespecified escalation pathways should be launched. Privacy-preserving analytics, ideally using federated evaluation, should be configured [21,22]. Billing workflows that map nurse-delivered digital activities to covered services should be established, with documentation standards to prevent overuse.

## Illustrative Nursing Workflow

### Scale With Guardrails (9‐18 Months)

Risk-tiered labeling and PCCPs for adaptive updates should be adopted. Only tools that meet certification criteria for appropriate design, content moderation, and transparency with fairness audits should be procured. Integration into measurement-based, stepped care pathways with clear tapering and exit criteria should be expanded. The federated RWE network should be activated, and quarterly dashboards with subgroup performance and safety signals should be published.

### Continuous Learning (≥18 Months)

Procurement and reimbursement criteria based on RWE should be recalibrated. Red teaming and child safety audits should be refreshed annually. PCCPs should be updated using drift monitoring and postmarket findings.

## Minimum Viable Implementation Considerations

### Overview

We emphasize that not all elements of the road map need to be implemented simultaneously. In resource-constrained school districts or community clinics, a minimum viable implementation may consist of clearly defined intended use and risk tiering, nurse-led human oversight with basic escalation pathways, and a small core set of safety and outcome indicators. More advanced components—such as federated evaluation networks or formal certification—can be layered in over time as institutional capacity grows.

### Evaluation Framework

For day-to-day operations, we recommend a short indicator set that most schools and clinics can track without major new infrastructure. In addition to chatbot-derived use and symptom data, this minimum dataset should include structured nursing data elements already generated in routine care—for example, triage category; follow-up completion; escalation timing; unsuccessful contact attempts; supervision time; and documented concerns related to sleep, functioning, or caregiver strain. At a minimum, programs should monitor access and equity, including uptake and retention and stratified outcomes by key demographic and socioeconomic proxies [17,18,23,24]. They should also monitor safety, including inappropriate content, time to escalation for crisis flags, adverse events graded by severity, and model rollback events. Clinical and functional outcomes should include validated ADHD symptom measures and, where feasible, school functioning, such as attendance and work completion alongside adherence to behavioral plans [1,2,11]. Because implementation succeeds or fails on acceptability and workload, programs should track youth and caregiver experience and nurse supervision burden. Finally, economic value should be assessed in practical terms, such as avoided no-shows or urgent visits, staff time reallocation, caregiver time saved, and net program costs relative to benefits.

Standardized nursing data may also serve as a practical bridge between governance and predictive intelligence: if captured consistently and audited appropriately, they can support subgroup monitoring; more tailored follow-up intensity; and earlier human review of clinically concerning patterns across clinic, school, and home settings.

### Financing and Procurement

In the near term, many systems can finance nurse-led digital care using existing codes—behavioral health integration or collaborative care for coordination and supervision and remote therapeutic or patient monitoring for structured data review—when services meet eligibility and documentation requirements. Procurement can leverage established assessment criteria to mandate safety, interoperability, evidence thresholds, and transparency artifacts such as model cards and data sheets [17,23,24]. For child-facing tools, age-appropriate design, content moderation, parental controls, and reliable crisis escalation should be required in the request for proposals [18-20]. Vendors that support federated evaluation and publish postmarket performance should be preferred.

### Ethical, Legal, and Social Considerations

Minors’ rights require developmentally appropriate consent, transparency, and parental controls. Content safety and age gating should be active prior to deployment, and crisis detection must escalate rapidly to human support. Data should be minimized and processed on the device when feasible, with auditable logs and role-based access. Because care traverses clinics and schools, compliance must distinguish HIPAA (Health Insurance Portability and Accountability Act)-governed health data from Family Educational Rights and Privacy Act–governed education records and set appropriate data sharing agreements [25,26]. Fairness should be treated as a practical obligation: subgroup performance should be documented, and disparities should be remediated with targeted outreach, assistive technologies, and human review for edge cases [17,18,23,24].

Regulatory examples drawn from the United States and Europe are presented as illustrative rather than prescriptive. The framework is meant to be adapted to local laws, workforce models, and cultural contexts—including Asia-Pacific settings—where the day-to-day reality may look different (eg, school nursing roles and staffing vary widely, and many systems rely more heavily on family- and community-based support than on school-based health infrastructure). In these contexts, the core requirements remain the same—clear intended use and risk tiering, practical child safeguards, and reliable human escalation—but the responsible human-in-the-loop lead may be a community nurse, primary care team, or another locally appropriate role rather than a US-style school nurse.

## Positioning the Nurse-Led, Risk-Tiered Framework Relative to Existing Governance Models

Existing digital health and AI governance frameworks—such as National Institute for Health and Care Excellence guidance, the Food and Drug Administration’s digital health policies, and the World Health Organization’s AI governance recommendations—provide essential high-level principles for safety, evidence generation, and risk management. However, these frameworks do not explicitly address how day-to-day oversight should be operationalized for minors with ADHD across clinical, school, and home settings. Our framework does not propose a new regulatory standard; rather, it translates these established principles into a practice-facing, nurse-led governance and implementation model. Its distinctive contribution lies in foregrounding (1) nurse-led human-in-the-loop supervision; (2) risk-tiered safeguards tailored to pediatric ADHD, including overreliance and crisis escalation risks; and (3) a concrete implementation road map that links regulation, reimbursement, and procurement with frontline workflows and outcome monitoring. In this way, the framework complements existing governance instruments by addressing gaps at the point of care where conversational agents are actually deployed.

For safety governance, the chatbot incorporates predefined crisis flag categories and escalation thresholds aligned with pediatric mental health practice. Over time, such rule-based safeguards may be complemented by cautiously implemented predictive review tools derived from standardized nursing data while nurse-led judgment remains primary. Crisis flags include but are not limited to explicit self-harm or suicidal language, acute emotional dysregulation persisting across multiple sessions, or repeated expressions of hopelessness unresponsive to in-chat de-escalation prompts. When a high-risk flag is triggered, the system initiates an alert to the designated nurse within a predefined time window (eg, immediate escalation for imminent risk and within 24 hours for high-risk but nonimminent flags), prompting nurse-led assessment and triage. Depending on severity, the nurse escalates care through established handoff pathways to physicians, child psychiatrists, school health professionals, or emergency services. This protocolized workflow ensures that the chatbot functions as an adjunctive early warning system rather than a stand-alone crisis intervention.

## Limitations

This Viewpoint has several limitations. First, the evidence base for AI-powered therapy chatbots specifically targeting ADHD in minors remains small, heterogeneous, and short in duration. Most studies evaluate feasibility or short-term symptom change in convenience samples, with limited replication; inconsistent outcome measures; and scarce reporting on school functioning, caregiver burden, or long-term adherence. Safety outcomes are also underreported; few studies use standardized harm taxonomies or prospectively capture inappropriate content, missed escalations, or overreliance.

Second, generalizability is uncertain. Usability, engagement, and effectiveness can vary by age, developmental stage, language and literacy, co-occurring conditions (eg, learning disabilities, anxiety, and autism), and social determinants (eg, connectivity and device access). Our equity recommendations are grounded in principle but require local tailoring and co-design with youth and caregivers to avoid unintended exclusion.

Third, policy and regulatory contexts are evolving and jurisdiction specific. Risk classification, the applicability of device regulations, and the operationalization of PCCPs differ across regions and may change rapidly. Similarly, reimbursement pathways for nurse-delivered digital care vary by payer and country, and our examples (behavioral health integration, remote therapeutic monitoring, and remote patient monitoring) may not apply universally. Procurement frameworks (eg, Digital Technology Assessment Criteria) also differ and may be updated over time.

Fourth, several elements of the framework are normative and have not yet been tested together as a complete package. We infer feasibility from adjacent digital health implementations and governance guidance, but the combined impact of risk-tiered regulation, certification and procurement criteria, financing mechanisms, and a federated RWE network remains to be demonstrated in prospective, multisite evaluations.

Fifth, important technical uncertainties persist. Adaptive models may drift; fairness audits and subgroup performance reporting are not standardized; and methods such as red teaming, age gating, and on-device processing need consistent benchmarks and transparent reporting. Interoperability and data minimization can be tense, and federated evaluation requires agreements and tooling that many organizations do not yet possess.

Finally, this paper does not address all legal and ethical issues exhaustively. We do not cover cross-border data transfers, vendor lock-in, or intellectual property constraints in depth, and we do not provide jurisdiction-specific legal advice. Readers should interpret the framework as a pragmatic starting point to be adapted with local legal counsel, clinical governance, and community input.

Despite these limitations, we argue that converging principles—risk-tiered regulation with PCCPs, age-appropriate design and transparency, financing for nurse-led integration, procurement aligned with safety and equity, and federated RWE—offer a credible pathway from cautious pilots to safe, equitable scale. Prospective, transparent evaluations that report benefits and harms by subgroup will be essential to refine and validate this approach.

## Conclusions

AI-powered therapy chatbots and virtual companions are likely to become a routine adjunct to ADHD care across clinics, schools, and homes. Their promising psychoeducation, coaching between visits, and structured inputs for measurement-based care will only be realized if deployment is paired with safeguards that are proportionate to risk and responsive to the needs of children and families. Nursing leadership is central to this task. As supervisors of day-to-day use, nurses can anchor human-in-the-loop oversight, ensure that escalation pathways work in practice, and help translate technical standards into workable routines.

A pragmatic agenda emerges from our analysis. First, risk-tiered governance for conversational agents, including PCCPs for adaptive models, should be adopted so that updates are transparent and reversible. Second, the real work of nurse-delivered digital care—triage, education, coaching, monitoring, and escalation—should be financed so that integration is sustainable rather than discretionary. Third, procurement decisions should be made based on clear, child-appropriate criteria that require age-appropriate design, content safety, transparency artifacts (eg, model cards and data sheets), interoperability, and fairness audits. Fourth, a federated RWE and safety surveillance network should be established using a shared harm taxonomy and standardized indicators so that benefits and harms can be tracked across settings without compromising privacy.

These steps are actionable now even as science matures. They can expand access for underserved youth; reduce unwarranted variation; and surface problems early, enabling course correction before harms accumulate. Equally importantly, they will generate the cumulative, subgroup-specific evidence that has been missing from the literature. With nursing leadership at the helm and with governance that matches risk to oversight, health systems and schools can move from cautious pilots to safe, equitable scale—bringing the advantages of well-designed conversational tools to the young people who stand to benefit the most. More broadly, recent nursing research suggests that structured nursing activity and diagnosis data can contribute to outcome prediction and early warning workflows in other care settings, supporting the future feasibility of hybrid oversight models in which standardized nursing data complement rather than replace nurse-led judgment [15,27].

## Abbreviations

ADHD: attention-deficit/hyperactivity disorder
HIPAA: Health Insurance Portability and Accountability Act
PCCP: predetermined change control plan
RWE: real-world evidence
